# Elucidation of the growth phenotypes of 82 strains from the *Halomonas* genus

**DOI:** 10.3389/fbioe.2026.1920469

**Published:** 2026-08-28

**Authors:** Alexander Antoine Maria Baumbach, Waritthorn Thanakarn, André A. B. Coimbra, Iona Wilkinson, Lanqi Zhang, Ivano Luigi Colao, Duygu Dikicioglu

**Affiliations:** 1 Department of Biochemical Engineering, University College London, London, United Kingdom; 2 The Manufacturing Futures Laboratory, University College London, London, United Kingdom

**Keywords:** exponential growth phase, Halomonas, log phase, maximum specific growth rate, microbial growth curve, non-model organism, optical density, phase of growth

## Abstract

**Introduction:**

Microbial growth phenotype is commonly summarised by a small set of parameters: specific growth rate, lag phase duration, maximum cell density, and growth curve profile; yet even within a single genus these characteristics can vary substantially across species, with direct consequences for how growth performance is interpreted and compared. This challenge is particularly pertinent for the largely uncharacterised *Halomonas* genus, whose members are increasingly proposed as next‐generation industrial biotechnology chassis owing to their halotolerance and capacity for open, non-sterile cultivation.

**Methods:**

In this work, the growth phenotypes of 82 *Halomonas* strains were assessed under collection‐recommended conditions of temperature, growth medium, and salt concentration using online optical density measurements in 96‐well plates.

**Results:**

Maximum specific growth rates ranged from 0.05 ± 0.03 to 1.14 ± 0.34 h^-1^ (mean 0.44 ± 0.22 h^-1^), with the shape of the growth curve frequently deviating from the canonical sigmoidal form and displaying substantial inter-strain variability. No meaningful correlation was identified between any growth parameter and either cultivation temperature or salt concentration (−0.39 ≤ ρ ≤ 0.39). Considered collectively, growth‐associated parameters identified *H. hamiltonii, H. zhaodongensis, H. heilongjiangensis*, and *H. magadiensis* as promising candidates for future biomanufacturing applications. The variability observed in growth curve profiles raised a critical methodological concern regarding researcher-dependent identification of growth phases and the computation of growth parameters. Six independent analysts assessed the same dataset, and at least one determined a significantly different maximum specific growth rate for half of the strains examined (p Maximum specific growth rates ranged from 0.05 ± 0.03 to 1.14 ± 0.34 h‐1 (mean 0.44 ± 0.22 h‐1), with the shape of the growth curve frequently deviating from the canonical sigmoidal form and displaying substantial inter‐strain variability. No meaningful correlation was identified between any growth parameter and either cultivation temperature or salt concentration (−0.39 ≤ ρ ≤ 0.39). Considered collectively, growth-associated parameters identified H. hamiltonii, H. zhaodongensis, H. heilongjiangensis, and H. magadiensis as promising candidates for future biomanufacturing applications. The variability observed in growth curve profiles raised a critical methodological concern regarding researcher-dependent identification of growth phases and the computation of growth parameters. Six independent analysts assessed the same dataset, and at least one determined a significantly different maximum specific growth rate for half of the strains examined (p < 0.05).

**Discussion:**

This analyst-dependent variation has broad implications beyond strain characterisation: analyst interpretation directly affects reported growth rates, which serve as key inputs and constraints in metabolic modelling and upstream bioprocess design. The reliability of both primary literature and secondary computational analyses built upon these data is therefore directly affected by analyst-specific decisions, a problem further compounded for poorly characterised organisms, whose growth parameters are often borrowed from related species in the absence of species-specific data, introducing a largely unacknowledged source of uncertainty. This foundational phenotypic dataset highlights an urgent need for standardised, generalisable frameworks for growth parameter determination, particularly for non-model organisms.

## 1 Introduction

Biotechnological applications have historically focused on the use of model organisms such as *Escherichia coli* and *Saccharomyces cerevisiae* as hosts, due to several practical reasons including ease of cultivation, fast growth rates and ability to achieve high cell densities, as well as the availability of effective genetic toolkits (Russell et al., 2017; Riley and Guss, 2021). Non-model organisms, which can demonstrate more diverse phenotypes and capabilities than traditional model organisms, have increasingly become a novel focus in engineering biology over the past decade. Such capabilities include the natural production of novel or high-value products (Ye and Chen, 2021; Pham et al., 2019), tolerance to environmental stresses (Ye et al., 2023; Zheng et al., 2025; Zhang et al., 2018), and the utilisation of alternative substrates and feedstocks (Riley and Guss, 2021; Yoshida et al., 2016).

Among non-model organisms, the *Halomonas* genus attracts increasing interest due to its halophilic properties and its native ability to produce ectoine and polyhydroxyalkanoates (PHAs). Its ability to survive in high salt concentrations is attractive due to the reduced risk of culture contamination in open and non-sterile fermentations with reduced energy costs and freshwater consumption (Zheng et al., 2025; Ye and Chen, 2021; Chen and Jiang, 2018).

Unsurprisingly, many non-model organisms remain poorly characterised. This is of particular importance for the *Halomonas* genus since a number of species in addition to the type species has been identified as promising candidates as novel engineering biology chassis. To this end, Mata and colleagues compared the growth characteristics and morphological, physiological, biochemical, nutritional and microbial susceptibility of 21 *Halomonas* species, reporting the high variability in the cultivation temperatures and salt tolerance of these species. Among those, some species were reported to be facultative anaerobes, whilst others lacked this ability (Mata et al., 2002). These findings further underscore the need for a comprehensive characterisation effort and an accompanying *Halomonas* repository, enabling researchers to identify the specific traits that render a given species suitable for their intended application.

Growth curves are commonly used to characterise the growth phenotypes of bacterial strains (Wiser and Lenski, 2015; Opalek et al., 2025; Ghenu et al., 2024; Stevenson et al., 2016). They typically follow a sigmoidal shape, consisting of 4 key stages: the lag phase, the exponential (also commonly known as log) phase, the stationary phase, and the death phase (Zhang et al., 2018). The phenotype of a bacteria is typically summarised by key parameters of the growth curve including the length of the lag phase, the maximum specific growth rate (*μ*_max_), the maximum cell density (also represented by the maximum optical density), and the shape of the growth curve (Zwietering et al., 1990; Ghenu et al., 2024; Monod, 1949). These key parameters allow scientists to understand bacterial strains, how they are affected by different environments or conditions, asses their fitness and identify suitable ones demonstrating desirable characteristics for a specific application (Mira et al., 2015; Hall et al., 2014; Wiser and Lenski, 2015; Ghenu et al., 2024). Phenotypical performance of a strain can also assist the identification of optimal conditions to increase the efficacy or the productivity for a target application.

Growth metrics such as *μ*_max_ and the maximum cell density are also essential inputs for computational models, which describe and predict the growth-associated metabolic behaviour of bacteria. These models simulate cellular behaviour under different conditions, such as nutrient limitation, and predict population response (Zwietering et al., 1990). Due to their analytical and predictive power, these models are frequently adopted to optimise fermentations and guide process scale-up (Du et al., 2022). The essential role of such models renders the provision of accurate growth parameter measurements as input imperative.

One of the most frequently estimated growth parameter is the growth rate (Ghenu et al., 2024). There are numerous methodologies used to estimate growth rates, from model-free methods such as the exponential estimation introduced by Monod, to a variety of different model-based methods (Monod, 1949; Ghenu et al., 2024; Zwietering et al., 1990; Mira et al., 2017). Indeed, even the definitions of the terms *μ* and *μ*_max_ can vary within the literature, causing confusion and, at times, leading to misuse (Ghenu et al., 2024; Perni et al., 2005).

An increasing number of new tools and methods were proposed for bacterial growth curve modelling in the past couple of decades. While their review and critical assessment is not within the remit of the current work, this question was recently addressed in a study by Ghenu et al. In their work, different methods developed for calculating *μ*_max_ were reviewed and four published datasets were re-analysed using a variety of different model-free and model-based methods. *μ*_max_ estimates were shown to differ vastly depending on the method used. Authors emphasised that there was no preferred method for calculating the maximum specific growth rates and that the best method may vary depending on the dataset. Model-based methods were reported to struggle when analysing data with high levels of noise or growth patterns that differ from the typical sigmoidal growth curves. In these instances, model-free methods were shown to demonstrate superior robustness (Ghenu et al., 2024). While variations in *μ*_max_ estimates due to method selection has been investigated by this recent study, the impact of the human decisions on these calculations and the subsequent variability introduced by the anthropogenic factors has not yet been investigated systematically.

There is a rising interest in the adoption of *Halomonas* species for bioprocessing applications. At time of writing, the List of Prokaryotic names with Standing in Nomenclature reports 130 species of *Halomonas* (Freese et al., 2026). However, the current literature reports physiological data focussed on a limited number of species, including but not limited to *H. elongata, H. bluephagenesis*, *H, boliviensis,* and *H. campaniensis*, leaving a large fraction of the *Halomonas* genus uncharacterised, with numerous strains unexplored. The first step towards the utilisation of any species in engineering biology is the elucidation of their growth phenotypes.

In this study the growth phenotypes of 82 strains of the *Halomonas* genus were assessed under culture collection-recommended conditions using optical density measurements (OD_600_) recorded in 96-well plates. We base our use of the term phenotype on the observable growth characteristics, which are determined by both the genotype of 82 different strains and of the environmental influences represented by 27 unique cultivation conditions. Five parameters related to the growth physiology: (i) The maximum specific growth rate, (ii) the duration of the lag phase, which will be referred as the ‘lag phase length’ from this point, (iii) the time that the culture takes to reach stationary phase, which will be referred as ‘time to stationary phase’ from this point, (iv) the maximum OD_600_, and (v) the shape of the growth curve, which will be referred as the ‘growth curve shape’ from this point, were determined for each strain. This was followed by a systematic investigation of the anthropogenic factors on the maximum specific growth rate calculations based on the analysis of 72 *Halomonas* strains by six independent researchers.

## 2 Methods

### 2.1 Strain procurement and stock library generation

Of the 82 *Halomonas* strains used in this study, 81 were from the DSMZ (German Collection of Microorganisms and Cell Cultures), and *H. eurialkalitoleranis* was from the ATCC (American Type Culture Collection). Growth conditions and media used were determined based on the recommendations from the DSMZ and ATCC websites in addition to further discussion with the DSMZ Team. Recommended growth media were supplemented with sodium chloride (Reagent Plus® ≥99%, Sigma-Aldrich, Cat. No. S9625) as recommended (Supplementary Table S1).

Strains recommended to be grown in DSMZ Media 514 (DIFCO 2216), were grown in either Marine Broth 2216 (Merck Millipore, Cat. No. 76448) or BD Difco^TM^ Dehydrated Culture Media: Marine Broth 2216 (BD, Cat. No. 279110) as indicated in Supplementary Table S1.

Strains were shipped freeze dried from the DSMZ, and frozen from the ATCC. They were processed as per the collection-specific processing protocols and grown in their collection-recommended growth conditions for 24h. Following this, they were pelleted by centrifugation at 4 °C and 4000RPM for a minimum of 20 min. The supernatant was removed and the cells were resuspended in 5 mL of fresh medium. The resulting culture was used to create beaded stocks in VWR Cryoinstant 2 mL Vials (Avantor VWR, USA) for long term storage at −80 °C. The remaining 3 mL was mixed with 600 µL of 100% glycerol to achieve a final glycerol concentration of 17%. This mixture was then aliquoted into sterile cryovials and immediately stored at −80 °C for short term use.

Any suspected contaminations or mixed cultures were addressed through a combination of methods and approaches including spot check visual inspection, microscopy imaging, gram staining and 16S rRNA sequencing. There were no contaminations nor any evidence of non-monoculture samples detected in the strains received from the culture collections.

### 2.2 Growth curve measurements

Growth curves were measured using CLARIOstar Plus microplate readers (BGM Labtech, Germany). The SMART control–CLARIOstar Plus and CLARIOstar Mars software was used to operate the microplate reader and for data recording.

For precultures, 100 μL of glycerol stocks of each strain was inoculated in 10 mL of its cognate collection-recommended medium and the cells were grown overnight (or longer if needed) in 50 mL vented capped centrifuge tubes at the respective strain-recommended temperature with 200 RPM agitation. A fresh 5 mL culture was prepared for each strain in a similar way, adjusting the starting OD_600_ of the culture to 0.05. This was aliquoted into 96 well plates (Sarstedt, Germany) at a volume of 200 μL per well, with a minimum of six replicates per strain, and fresh medium in four wells as negative controls. Strains with the same recommended growth temperatures were grouped and placed into the same 96 well plate to reduce the total number of runs required. Plates were then sealed with the AeraSeal^TM^ Film (Excel Scientific, USA), which is an air-permeable sealing membrane.

The 96-well plates were placed in the plate reader and incubated at the strains’ recommended temperatures with 200 RPM double orbital shaking. Central OD_600_ readings were taken with 50 flashes every 5 min. Overflow OD_600_ readings were set to a value of 2.5. For those strains that were cultivated for longer than 83 h, readings were taken every 10 min instead, to extend the culture data recording timeframe.

Prior to large scale analysis, the positioning effects were tested by running 45 replicates of the same *Halomonas* sp. together growing under the same condition, with the remaining wells left as control. This experiment was repeated twice to test inter-plate consistency across the findings. Three types of edge contact were identified: (i) wells located at the periphery, (ii) adjacent wells to those at the periphery and (iii) those located at the innermost centre of the plate. Comparing the growth metrics for the wells positioned at (i) vs. (ii), (i) vs. (iii), and (ii) vs. (iii) for either plate did not yield any significant differences (all p-values >0.05).

### 2.3 Growth curve analysis, characterisation, and growth metric calculations

Growth curve data was processed using RStudio (Posit Team, 2024). An offset was calculated per well so that the first OD_600_ reading was equal to the known initial OD_600_ value of 0.05. This per well offset was then applied to all readings. The processed OD_600_ data was then imported into Microsoft Excel where OD_600_ and log transformed optical density (ln (OD_600_)) were plotted against time (h) to analyse the growth parameters. Negative OD_600_ values caused by offset adjustment were omitted from further analysis. The growth curve patterns were characterised into one of 5 characterisation groups: sigmoidal shaped (S), double exponential growth (DG), M-shaped (M), atypically shaped (AS), or with multiple characteristics (MC).

Lag phase length was classified into one of three groups. Short lag (SL) for a lag phase between 0–5 h, intermediate lag (IL) for 5–10 h, and long lag (LL) for lag phases that were longer than 10 h. Lag phase length was characterised based on visual inspection of the mean of the replicates of the ln (OD_600_) *versus* time graphs for each strain.

The maximum specific growth rate (*μ*_max_) was calculated by first identifying the exponential growth phase from the linear range of the ln (OD_600_) *versus* time graphs by visual inspection. Atypical growth curves were accounted for by selecting the initial range of steepest linear growth with a minimum of 12 data points. The maximum number of data points that could be included in the calculation were included for each growth curve, which varied demonstrably across different strains (12–481 data points included). Linear regression was performed on the selected data points from the exponential growth phase, and the slope of the fitted line was reported as *μ*_max_. If multiple exponential growth regions were identified, such as those present in curves exhibiting DG growth characteristics (see Section 3.5), the initial exponential growth phase was considered to represent growth under the strains preferred growth conditions and nutrient availability. Therefore, the first region was selected for *μ*_max_ calculations.

Maximum OD_600_ was calculated by taking the maximum value recorded in each well. If singular data points demonstrating extreme and non-recurring anomalies were identified, the reading recorded as the maximum OD_600_ was manually adjusted by selecting the next highest reading record for that well.

The Time to Stationary phase (TtS) was calculated on pre-processed data: raw data was smoothed using RStudio (Posit Team, 2024) by calculating a rolling mean with a range of 13 data points. The TtS was taken as the time elapsed between inoculation and the start of the stationary phase. TtS is a particularly useful metric to assess strains, which show atypical growth patterns, or biphasic growth. The start of the stationary phase was then manually selected by visual inspection based on both the smoothed and raw data graphs. For strains showing multiple exponential growth phases, the first stationary phase following the initial exponential growth was selected provided that the cessation of growth could be confirmed.

### 2.4 Specific growth rate calculation comparison study

Data for 72 strains was shared with six independent researchers who were provided with the same set of instructions to analyse and calculate the specific growth rate for each strain. *μ* was used in the instructions rather than *μ*_max_ to allow flexibility for researcher specific interpretations for growth rate calculations. The data sent to researchers was the same data used for the growth phenotyping analysis except for *H. gomseomensis, H. ilicicola, and H. utahensis*, for which data from additional replicate runs were provided. Researchers were asked to manually select the exponential growth phases for these calculations, without the use of models or curve fitting. Data files for each strain were numbered to ensure that strain identity remained anonymous to researchers. The instructions provided to the researchers and the methodology that each researcher provided to complement their analysis is provided in Supplementary Methods.

### 2.5 Statistical analysis

Mean values, standard deviation (SD), and the coefficient of variation (CV, reported typically as percentage) were calculated for *μ*_max_, maximum *OD*_*600*_, and TtS.

Spearman’s ranked correlation coefficients were calculated using RStudio (Posit Team, 2024). Spearman’s rank correlation coefficients were interpreted as very weak (|*ρ*| ≤ 0.19), weak (0.20 ≤ |*ρ*| ≤ 0.39), moderate (0.40 ≤ |*ρ*| ≤ 0.59), strong (0.60 ≤ |*ρ*| ≤ 0.79), very strong (|*ρ*| ≥ 0.80).

To compare strains across the genus, *z*-score values were calculated for each metric (Microsoft Excel) with the assumption that the mean values and the CVs for each metric were samples drawn from a normally distributed population. For each strain, the mean *z*-score across all metrics was calculated to provide an overall comparative score relative to the genus represented by the average of these 82 strains. For metrics where lower values are often considered more advantageous, such as TtS and variability (CV) of each metric, the *z*score was multiplied by −1. To standardise the variability and allow a cross-species comparison, the CV was used for each metric rather than the SD. Two-tailed significance thresholds were determined using the standard normal distribution, with thresholds of |z| > 1.645, |z| > 1.96, and |z| > 2.575 corresponding to p < 0.10, p < 0.05, and p < 0.01, respectively.

The effect that different scientists may have on specific growth rate calculations was investigated using a linear mixed-effects modelling approach. Models were implemented in R (version 4.4.0) (Posit Team, 2024) using the *lme4* (v.1.1.35.3), *lmerTest* (v3.2.1), and *emmeans* (v2.0.2) packages (Bates et al., 2015; Kuznetsova et al., 2017; Lenth and Piaskowski, 2026). Specific growth rate was modelled as a function of strain, analyst, and their interaction. Replicate datasets were included as a random effect to account for natural variation between replicate growth curves within the same strain.

Significance of fixed effects was assessed using analysis of variance (ANOVA). Post hoc pairwise comparisons were performed using estimated marginal means with Tukey-adjusted p-values. Statistical significance was assessed at α = 0.05.

## 3 Results

A variety of growth recommendations were provided by DSMZ and ATCC to ensure the robust growth of all 82 *Halomonas* strains. Eight different types of media were recommended to support the growth of these strains. The recommended growth temperature and salt concentration ranged from 22 °C to 40 °C, and from 2% to 20.2% (w/v), respectively. The strains demonstrated varying levels of fitness under these conditions. *H. janggokensis* growth was recognisably slow, reaching an *OD*_*600*_ of 0.39 after 6 days of cultivation in 10 mL cultures, but yielding minimal identifiable growth when cultured in microwell plates, and was consequently removed from further data analysis.

While the recommended environment for *H. eurialkalitoleranis* culturing is reported as dark, the strain was able to grow in the absence of this restriction confirming light insensitivity. Upon correspondence with and subsequent confirmation of the research group who has submitted the strain to ATCC, a decision was made to exclude this culture collection recommendation from the experimental protocols. ATCC has been informed of this modification and the related correspondence for relevant correction.

### 3.1 Classification of *Halomonas* growth into characteristic curve patterns

**FIGURE 1:**
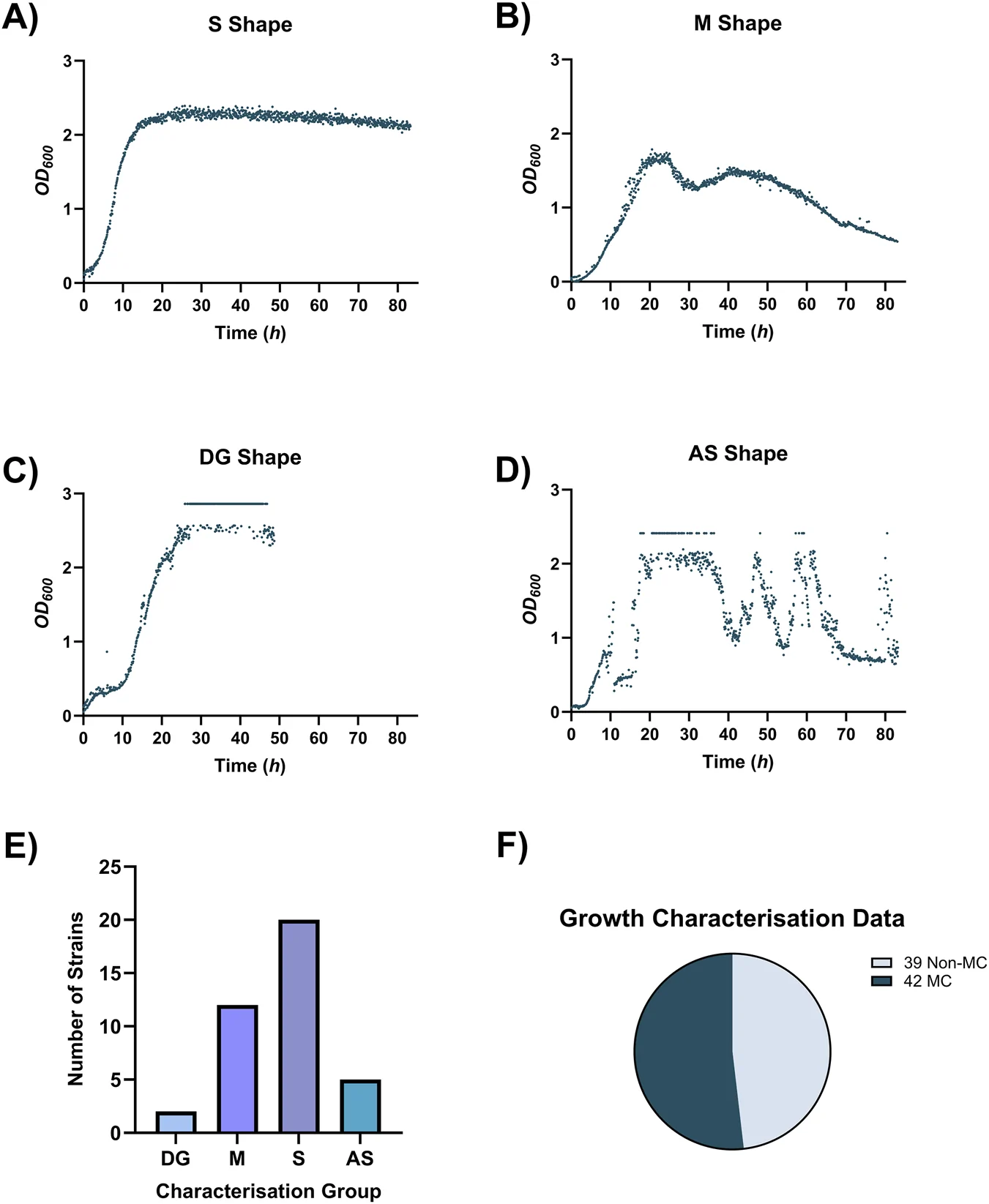
Overview of Growth Pattern Characterisation. **(A)** An example growth curve showing the sigmoidal growth patterns typical of bacterial growth shown in the (S) characterisation group. The growth curve shows a single replicate run for *H. halocynthiae.* **(B)** An example growth curve showing the decrease in cell density followed by an increase in the stationary phase, forming the growth patterns seen in the (M) characterisation group. The growth curve shows a single replicate run for *H. shengliensis.* **(C)** An example growth curve showing the biphasic growth patterns seen in the (DG) characterisation group. The growth curve shows a single replicate run for *H. alimentaria.* **(D)** An example growth curve showing the highly erratic growth patterns seen in the (AS) characterisation group. The growth curve shows a single replicate run for *H. nigrificans.* All growth curves were collected from strains grown in their recommended growth conditions, with OD_600_ recordings taken every 5 min. Time is shown in hours (*h*). **(E)** Bar chart representing the number of strains in the S, M, DG, and AS growth characterisation groups. **(F)** Pie chart showing the number of strains presenting multiple growth characteristics within their replicate runs (MC group), compared to non-MC strains that could be classified into one of the growth characterisation groups. Out of 81 strains, 42 (52%) strains were characterised into the MC group.

The 81 strains of *Halomonas* exhibited a variety of different growth patterns that, at times, differed from the canonical sigmoidal bacterial growth characteristics. Based on the patterns observed the growth curves were characterised into one of five groups: S, DG, M, AS, or MC, examples of which are provided in Figure 1. Group S displays a sigmoidal curve typical of bacterial growth (Figure 1A). M shaped curves are characterised by a decrease in optical density readings followed by an increase in the stationary phase to form an M shape instead of the typical flat trend (Figure 1B). Strains, which show multiple periods of decreased and increased cell density in the stationary phase were also classified into this group. DG shows biphasic growth patterns with a slowing of growth or a temporary stationary phase, followed by further growth or increase in OD that is sustained over multiple time points and visually distinct from noise (Figure 1C). AS shows Atypical S shaped curves (Figure 1D). Whilst these may still maintain a roughly or broadly attributable S shape, they are highly erratic curves and are difficult to classify into other groups. Finally, strains in group MC show multiple characteristics between repeats and could not be categorised into a single growth characteristic.

**FIGURE 2:**
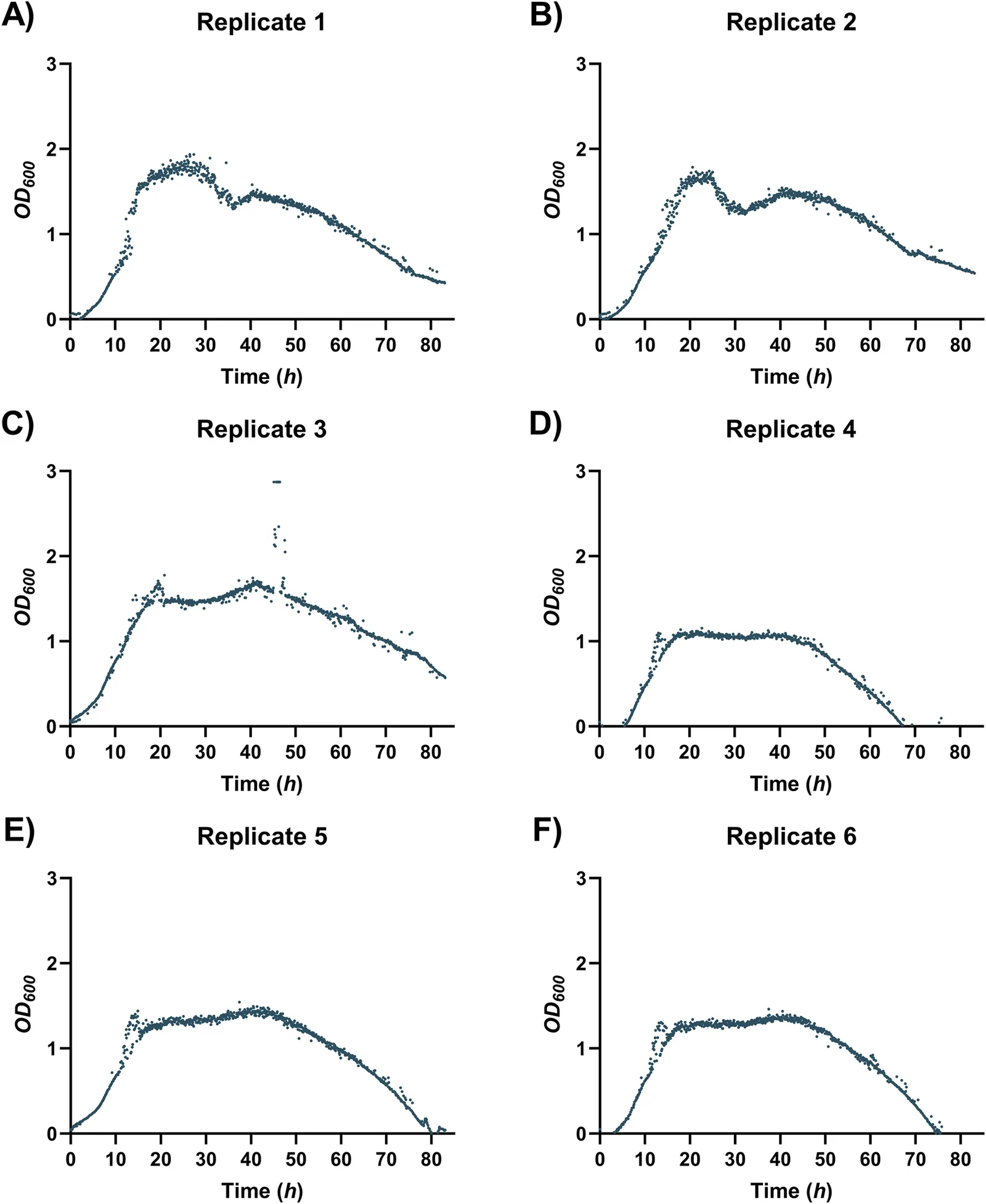
MC group growth curve characterisation example. Panels **(A–F)** display the growth curves for replicate runs 1-6 of *H*. *shengliensis*, respectively. *H*. *shengliensis* was cultured in its recommended growth conditions with OD_600_ readings recorded every 5 min. Time is shown in hours (h). *H*. *shengliensis* was classified into the MC growth characterisation group. Replicates 1 **(A)**, 2 **(B)**, and 3 **(C)** present an M shaped growth curve. However, replicates 4 **(D)**, 5 **(E)**, and 6 **(F)** are aligned with the typical sigmoidal S-shaped growth curve.

Overall, 48% of the strains (39/81) displayed consistently the same pattern across all replicates. Among these, 20 strains showed an S shape pattern typical of sigmoidal bacterial growth. The remaining DG, M, and AS groups represented the growth patterns of 2, 12, and five strains, respectively (Figure 1E). The remaining 42 strains were observed to follow MC-type behaviour with replicate runs demonstrating different growth characteristics (Figure 1F). An example of such a strain was *H. shengliensis*, for which, two replicate runs show a clear M shape (Figures 2A,B), whereas replicate 3 displays a less pronounced M shape characteristic, manifesting as a near M-S hybrid (Figure 2C). On the other hand, replicates 4, 5, and 6 show growth that aligns with the typical sigmoidal S shape followed by a death phase (Figures 2D–F).

**FIGURE 3:**
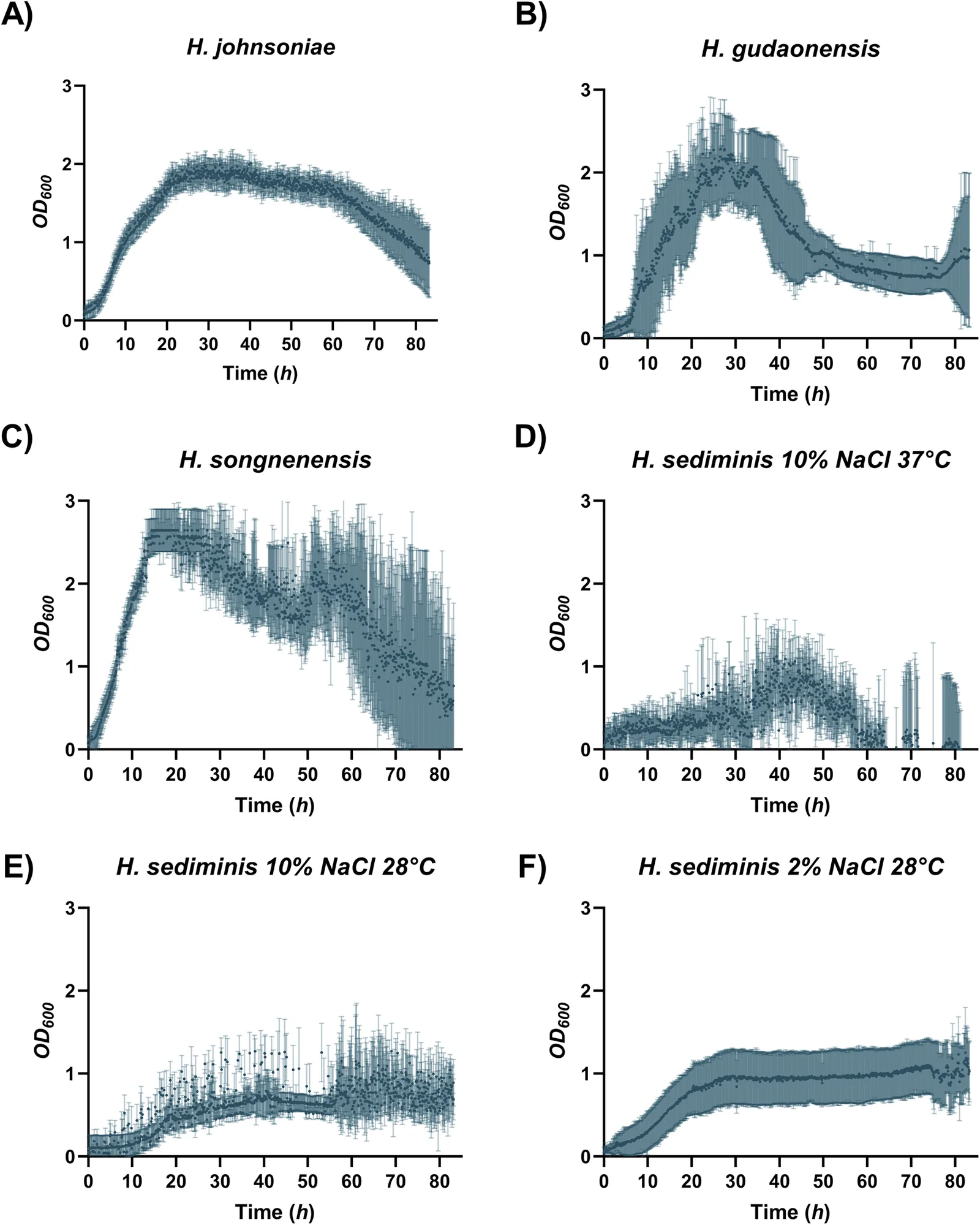
Variability of *Halomonas* growth curve profiles. All strains were grown in their recommended growth environment unless stated otherwise. **(A)** displays the mean growth curve for *H. johnsoniae* with error bars representing ±1 SD (n = 6), showing minimal variation across replicates. **(B)** displays the mean growth curve for *H. gudaonensis* with error bars representing ±1 SD (n = 6), showing high variation across replicates. **(C)** displays the mean growth curve for *H. songnenensis* with error bars representing ±1 SD (n = 6), showing high regional variation across replicates after the late exponential phase, centred around the stationary, and the death phases. **(D**-**F)** display the mean growth curve for *H. sediminis* grown in 10% NaCl at 37 °C **(D)**, 10% NaCl at 28 °C **(E)**, and 2% NaCl at 28 °C **(F)**, respectively. Error bars represent ±1 SD (n = 6). Panels demonstrate the progressive reduction in variability and the fluctuations in consecutive measurements upon modification of the culture settings.

Even among those strains that displayed the same growth curve shape among its replicates, variability in growth behaviour was prominent for some strains. An example of this may be observed in the comparison of the growth profiles of *H. johnsoniae* and *H. gudaonensis* (Figures 3A,B, Supplementary Figures S1, S2). *H. johnsoniae* displays minimal variability indicated by the relatively low standard deviation around the mean OD_600_ readings across the six replicate runs, whereas the variability was considerably high for *H. gudaonensis*. This is evidenced by the maximum CV for *H. gudaonensis*, which was 270% whereas the maximum relative standard deviation for *H. johnsoniae* remained as low as 88%. For other strains, such as *H. songnenensis*, the extent of variability increased towards late exponential and stationary phases, despite very low variability in early phases of growth (Figure 3C, Supplementary Figure S3).

*H. sediminis* showed suboptimal growth performance, visible in the form of snowflake-like aggregates, when cultured in the recommended medium (BD 514 supplemented to 10% salt concentration at 37 °C). This led to the plate reader detecting minimal identifiable growth with high fluctuations and spikes in *OD*_*600*_ readings (Figure 3D, Supplementary Figure S4). The original study initially characterising this strain reported growth in a temperature range of 4 °C–35 °C, and an optimum salt concentration of 2%–4% (Huang et al., 2008). While the reduction of culture temperature to 28 °C alleviated some of the challenges observed (Figure 3E, Supplementary Figure S5), reducing the salt concentration from the recommended 10% down to 2% yielded the most consistent growth pattern (Figure 3F, Supplementary Figure S6). Altering of the recommended growth conditions of *H. sediminis* to 28 °C and in 2% NaCl, and the cognate data were also shared with DSMZ.

The duration of the lag phase of each strain was classified into one of three groups; Short Lag (SL), Intermediate Lag (IL), or Long Lag (LL), in which the strains entered exponential phase after 5h, 5–10h, or >10h, respectively. Broadly speaking, 86% of strains were associated with a SL. The IL and LL groups included 8% and 6% of the collection, respectively.

### 3.2 Maximum specific growth rate, maximum attainable optical density and time taken to reach stationary phase as key growth metrics of *Halomonas*

To further elucidate the growth phenotypes of the *Halomonas* genus, three key mean metrics and their CVs $\left(n\geq 6\right)$ were calculated for each strain: the specific growth rate (*μ*_max_), the maximum *OD*_*600*_, and the time taken to reach the stationary phase (TtS). CV normalises the measured variability and consequently was selected in this study as a more suitable metric to assess variability across the genus than SD (Supplementary Table S2).

**FIGURE 4:**
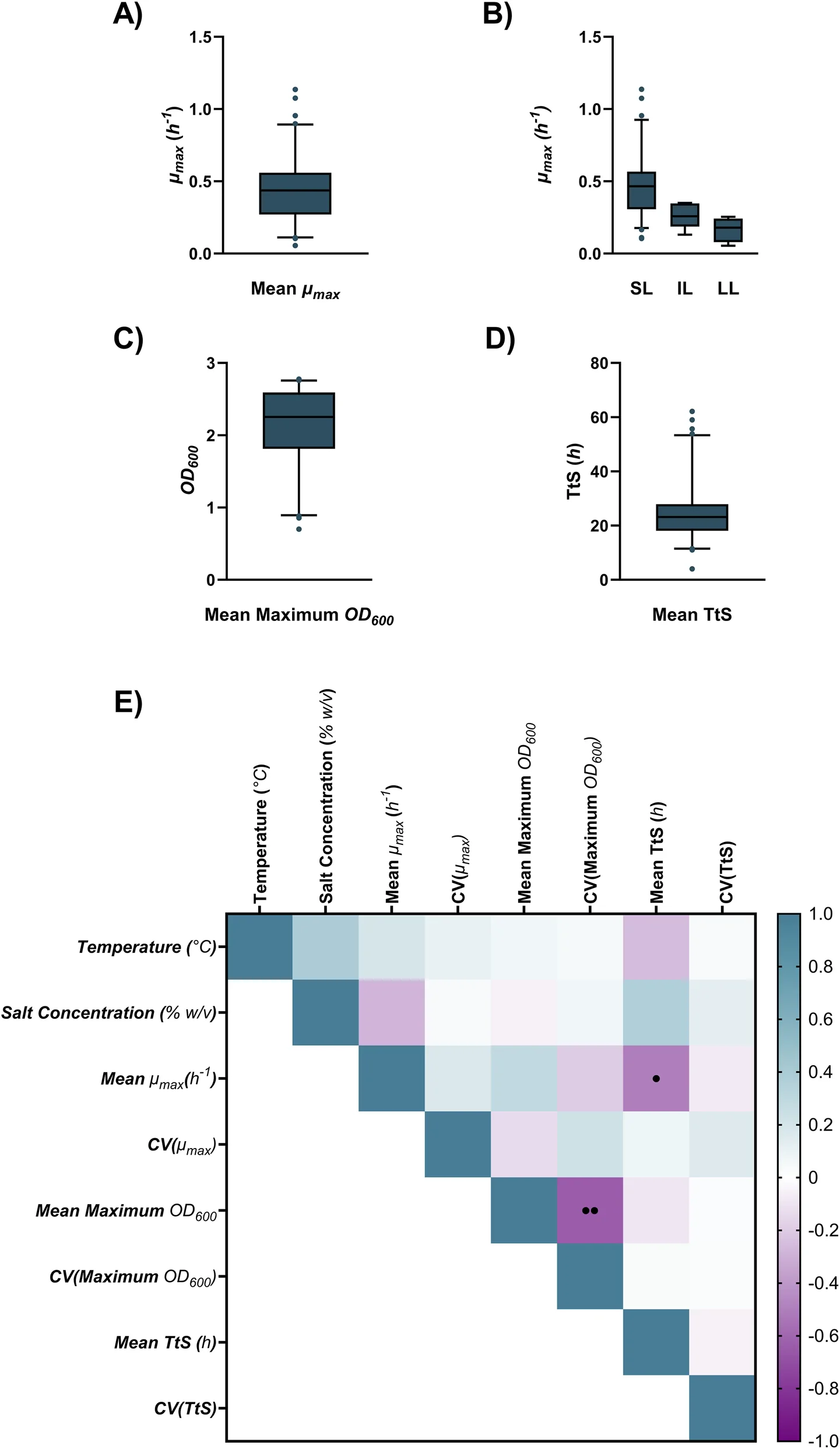
Overview of the calculated *Halomonas* growth metrics across 81 strains and their correlations. **(A)** Box and whisker plot showing the spread of mean *μ*_max_ (h^-1^) across the culture collection. **(B)** Box and whisker plot showing the spread of the mean *μ*_max_ (h^-1^) values of the strains in the small lag (SL), intermediate lag (IL), and long lag (LL) groups. The plots show a trend suggesting that strains with a longer lag phase have a slower mean *μ*_max_ (h^-1^). **(C)** Box and whisker plot showing the spread of mean maximum OD_600_ across the culture collection. **(D)** Box and whisker plot showing the spread of mean time to stationary (TtS) (h) across the culture collection. **(E)** Spearman correlation heatmap illustrating correlations between temperature, salt concentration, mean *μ*_max_ (h^-1^), mean maximum OD_600_, mean time to stationary phase (TtS) (h), and the cognate CV values for each metric. The legend shows the colours corresponding to calculated *ρ* values ranging from a strong positive correlation (+1) to a strong negative correlation (−1). A single black filled circle indicates moderate correlation |*ρ* | ≥ 0.4, and two black filled circles represent strong correlation |*ρ* | ≥ 0.6. No meaningful correlation (−0.39 ≤ *ρ* ≤ 0.39) was observed except for two cases: between (i) mean *μ*_max_ (h^-1^) and mean TtS (h), and (ii) mean maximum OD_600_ and ${CV}_{{maxOD}_{600}}$ , which showed moderate negative and strong negative correlations, respectively.

The mean *μ*_max_ of *Halomonas* strains was 0.44 ± 0.22 h^-1^ with values ranging from 0.05 ± 0.03 h^-1^ to 1.14 ± 0.34 h^-1^. Since the growth metrics were not available for an acceptably high number of strains in this work, an investigation of the population distribution was considered useful. The interquartile range (IQR) for *μ*_max_ spanned from 0.27 h^-1^ to 0.56 h^-1^ (Figure 4A). The fastest growing strains were *H. zhaodongensis, H. magadiensis,* and *H. sedimenti*, with a mean *μ*_max_ of 1.14 ± 0.34 h^-1^, 1.08 ± 0.45 h^-1^, and 0.95 ± 0.41 h^-1^, respectively. On the other hand, *H. gomseomensis, H. subglaciescola,* and *H. halmophila* were the slowest growing strains, with a mean *μ*_max_ of 0.05 ± 0.03 h^-1^, 0.10 ± 0.05 h^-1^, and 0.10 ± 0.05 h^-1^, respectively. The mean ${CV}_{\mu_{\max}}$ of the collection was 39% ± 16%, *H. caseinilytica* displaying the largest variation in calculated *μ*_max_ values, with a ${CV}_{\mu_{\max}}$ of 74%, whilst the most consistent *μ*_max_ values were determined for *H. venusta* with a ${CV}_{\mu_{\max}}$ of 11%.

Strains with a relatively longer lag phase had lower *μ*_max_ than those strains identified with short lag phases (Figure 4B). However, this broad generalisation should be assessed carefully since IL and LL groups only have 6 and 5 members, respectively, whereas 70 strains belong to the SL group; the unequal sample group sizes may affect the robustness of this conclusion.

The mean maximum *OD*_*600*_ was 2.13 ± 0.57 with values ranging from 0.70 ± 0.27 to 2.78 ± 0.21. The IQR covered 1.81 to 2.59. The maximum *OD*_*600*_ showed reduced variation in comparison to the *μ*_max_, with a mean ${CV}_{{maxOD}_{600}}$ of only 13% ± 7%. The highest variation was recorded for *H. gomseomensis* (39%) whereas the lowest variation was determined for *H. axialensis* and *H. eurihalina*, both with a ${CV}_{{maxOD}_{600}}$ of 3% (Figure 4C).

The strains in the collection had a mean TtS of 25 ± 11 h, with the IQR spanning 18–28 h. However, the TtS of strains such as *H. maura* and *H. gomseomensis* were as long as 59 ± 0 h and 62 ± 9 h, respectively. The extent of variation $\left({CV}_{TtS}\right)$ differed across strains, ranging from 0% to 48% (Figure 4D).

Spearman’s rank correlation coefficients (*ρ*) were calculated in order to investigate any correlations between growth metrics, as well as between the growth metrics and either the cultivation temperature or the salt concentration of the medium (Figure 4E, Supplementary Table S3). The only statistically meaningful correlations were identified between mean *μ*_max_ and mean TtS $\left(\rho =-0.5\right)$ or between mean maximum *OD*_*600*_ and ${CV}_{{maxOD}_{600}}$ $\left(\rho =-0.64\right)$ . Interestingly, no meaningful correlations were identified between the growth metrics and the cultivation parameters emphasising a level of diversity and unpredictability of phenotypical responses to the environmental variability across the *Halomonas* genus.

**FIGURE 5:**
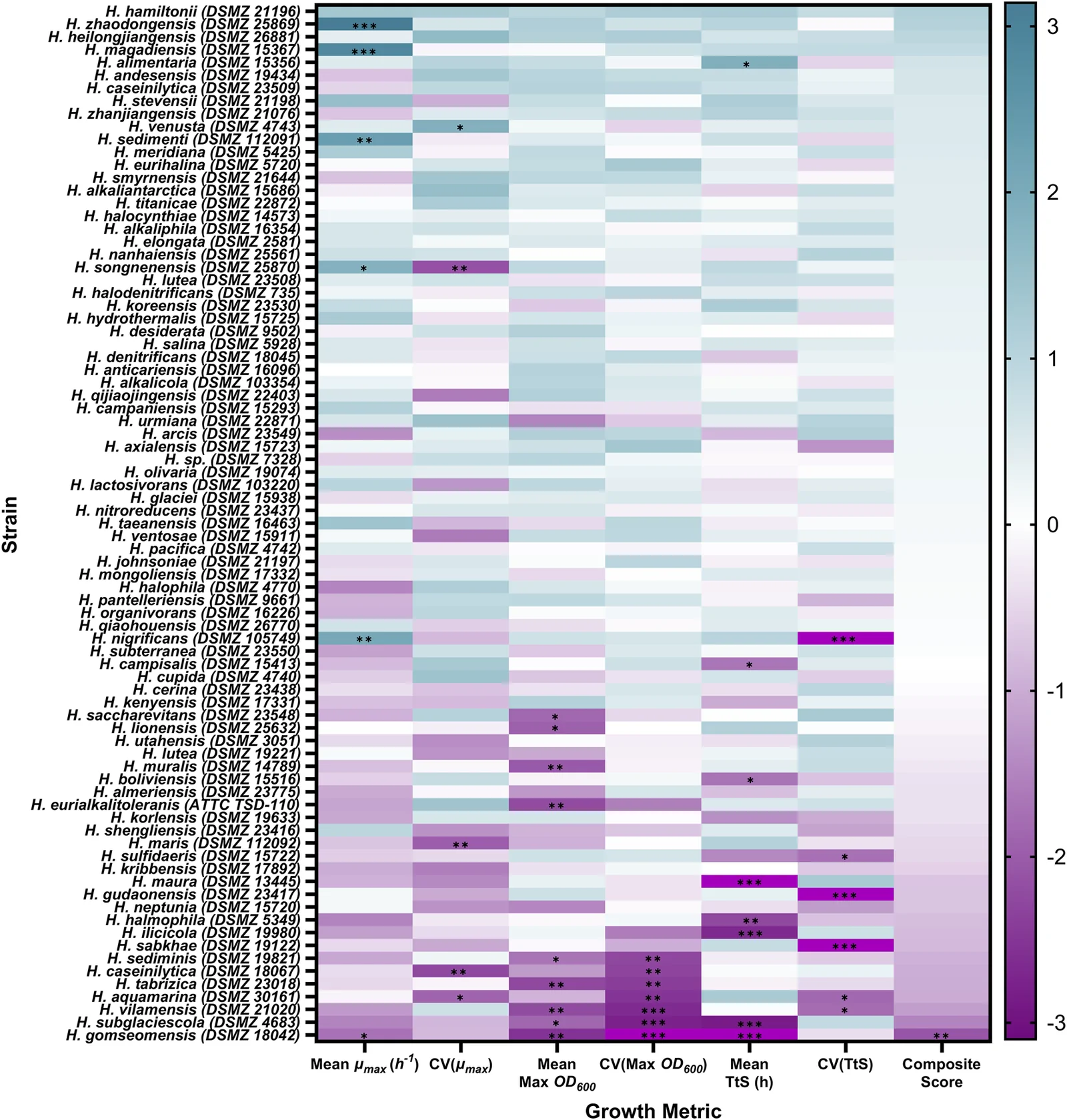
Comparative heat map of growth metric *z-*scores across the *Halomonas* genus. A heatmap of the *z*-scores for the mean *μ*_max_ (h^-1^), maximum OD_600_, and Time to Stationary (TtS) (h), and for their cognate CVs. The colour gradient on the legend represents the *z*-scores covering more than 99.7% of the Gaussian probability distribution. A composite score was calculated for each strain by taking the mean of all *z-*scores. Strains are ranked by the composite score, with the highest scoring strains placed at the top of the heat map. Two-tailed significance thresholds were determined using the standard normal distribution, with thresholds of |z| > 1.645, |z| > 1.96, and |z| > 2.575 corresponding to p < 0.10 (*), p < 0.05 (**), and p < 0.01 (***), respectively.

For systematic cross-strain comparison, z-scores were calculated for the mean value of these growth metrics and their corresponding coefficient of variation (CV). For metrics where lower values are considered advantageous such as CV, where lower values reflect greater stability, and TtS, where lower values indicate faster growth, z-scores were multiplied by (−1). This normalisation approach facilitates visual comparison and allows metrics measured on different scales to be combined without concern for differences in absolute values. A composite score was subsequently derived from all z-scores to reflect how each strain performs while taking into account CV-derived z-scores as an indicator of strain robustness (Figure 5, Supplementary Table S4).

**FIGURE 6:**
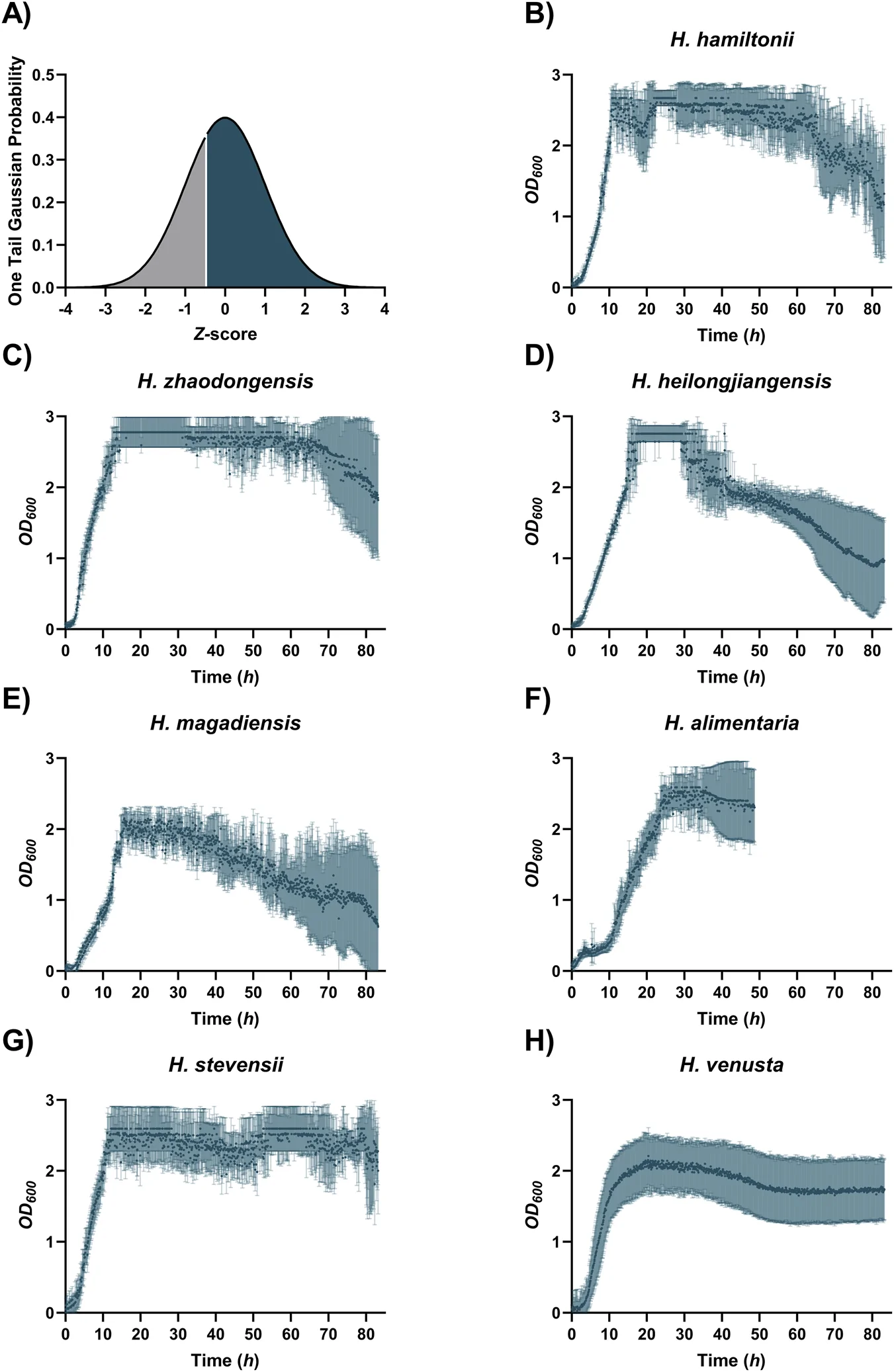
Growth curves of shortlisted candidate strains. The strains with the top ten highest composite z-scores were further shortlisted using a *z*-score threshold of −0.5 for mean *μ*_max_ (h^-1^), maximum OD_600_, and Time to Stationary (TtS) (*h*), yielding 7 shortlisted strains. **(A)** Shows a standard normal distribution curve highlighting the one-tailed probability corresponding to the *z*-score threshold of −0.5. The area highlighted in blue and grey represents 69.15% and 30.85% of the total probability, respectively. **(B–H)** Mean growth curve plots for *H. hamiltonii* **(B)** *, H. zhaodongensis* **(C)** *, H. heilongjiangensis* **(D)** *, H. magadiensis* **(E)** *, H. alimentaria* **(F)** *, H. stevensii* **(G)** *, and H. venusta* **(H)**, respectively. Strains were grown in their recommended growth conditions with OD_600_ readings recorded every 5 min. Error bars represent ±1 SD (n = 6).

From the top ten composite-scoring strains, a secondary threshold of (−0.5) was applied to individual z-scores, ensuring that shortlisted candidates performed no worse than approximately 69% of the genus population in any individual contributing metric (Figure 6A). When this threshold was applied to both growth metric and variability z-scores, four strains were shortlisted: *H. hamiltonii* (Figure 6B, Supplementary Figure S7), *H. zhaodongensis* (Figure 6C, Supplementary Figure S8), *H. heilongjiangensis* (Figure 6D, Supplementary Figure S9), and *H. magadiensis* (Figure 6E, Supplementary Figure S10). Relaxing the threshold to apply only to growth metric z-scores, excluding variability, expanded the shortlist by a further three strains: *H. alimentaria* (Figure 6F, Supplementary Figure S11), *H. stevensii* (Figure 6G, Supplementary Figure S12), and *H. venusta* (Figure 6H, Supplementary Figure S13).

One important insight emerging from this analysis is the considerable variability observed in growth curve shapes across the genus, and the strong dependence of specific growth rate calculations on curve morphology and how it is interpreted and handled by the researcher. This introduces a meaningful scientist-dependent source of variability in reported maximum specific growth rates. The following section presents our investigation into this researcher effect and its impact on the calculation of this key growth metric.

### 3.3 The analyst effect on specific growth rate determination

Six researchers were asked to calculate growth rates for the same dataset in order to investigate the extent to which µ_max_ calculations were influenced by the choice of the analyst. Specific growth rate, *μ*, was intentionally used in the instructions rather than *μ*_max_ to allow for researcher specific interpretations for growth rate calculations. They were sent the OD_600_ recordings for a minimum of six replicate runs for each one of the 72 strains and they were asked to independently analyse the data and calculate *μ* values for each replicate of each strain (Supplementary Tables S5, S6).

They were allowed flexibility to adopt their own methods provided that the exponential growth phase was selected manually, not through the use of computational models and line fitting methods. They were asked to maintain a consistent methodology throughout and provide a clarification for their decision concerning any specific data or in the identification of the exponential phase as required (Supplementary Methods 2.1-2.6). Five scientists calculated the *μ*_max_ using the classical exponential approximation method based on Monod growth kinetics. Exponential regions were manually identified by visual inspection identifying the linear range of the ln (*OD*_*600*_) vs. time graphs to provide the estimate of *μ*_max_ (Ghenu et al., 2024; Monod, 1949). Analyst #5 used non-modelling and non-line fitting based computational methods to calculate *μ*_max_ for every possible time point and time window and then selecting the *μ*_max_ based on subsequent filtering.

The use of computational methods considerably reduced the time taken to analyse the large dataset, yielding results in the timeframe of only a few hours to calculate all *μ*_max_ values. However, in some instances the *μ*_max_ estimations were disproportionally large compared to other results. Computational methods delivered a mean *μ*_max_ of 1.93 ± 2.08 h^-1^ for *H. subglaciescola*, a mean *μ*_max_ value ranging from 0.08 ± 0.07 to 0.11 ± 0.02 h^-1^ was reported in the remaining five analyses. Further investigation into the computationally facilitated approach showed that even post-filtering, the *μ*_max_ was selected from a region corresponding to the death phase of the growth curve (Supplementary Figure S14). The sporadic and atypical data recordings in the death phase leading to non-meaningful increases in OD_600_ were captured by the computational methodology and were mistaken to be representative of the exponential growth phase.

**FIGURE 7:**
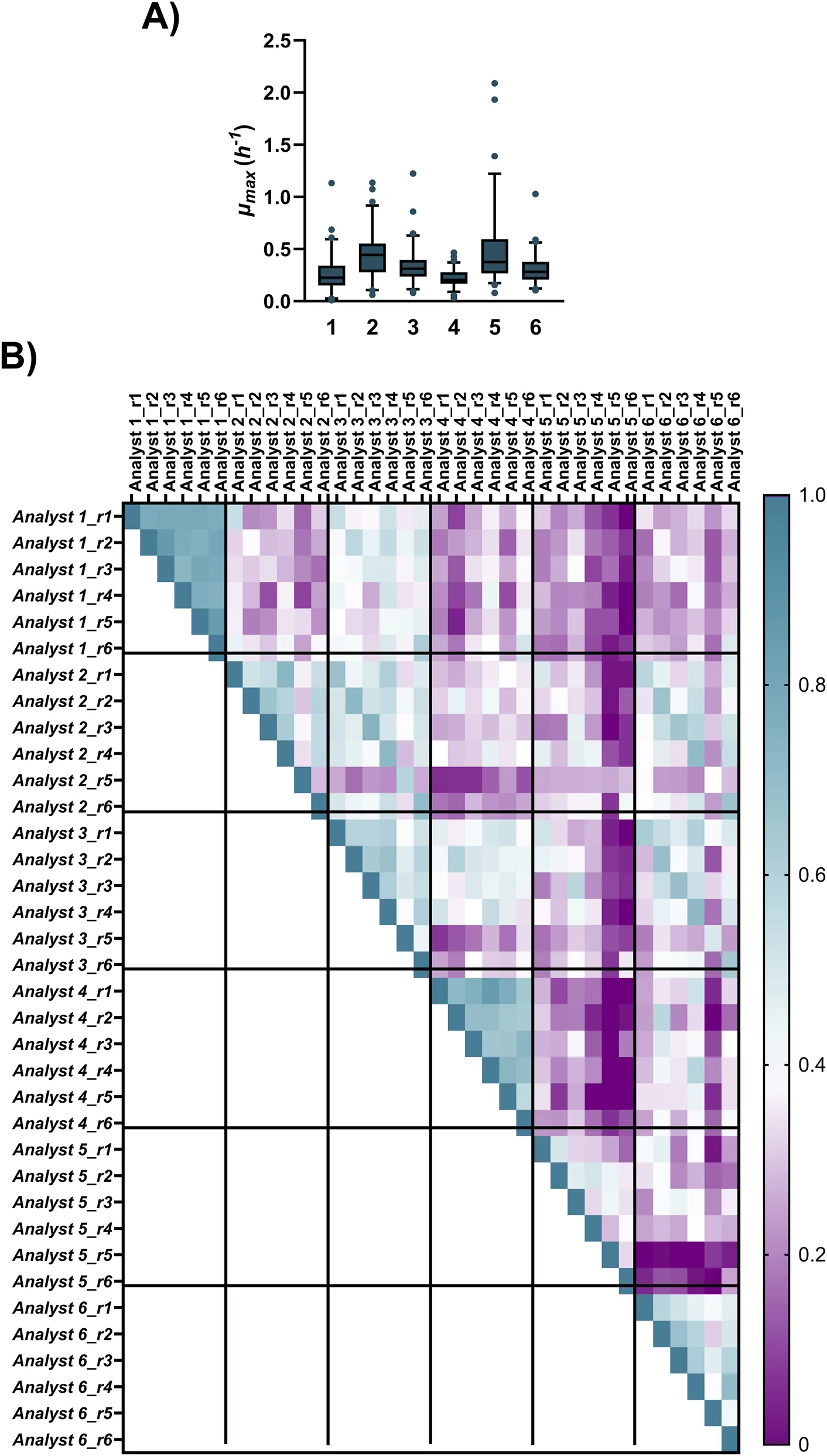
Overview of inter-analyst variation. **(A)** Box and whisker plots representing the spread of calculated *Halomonas* strain mean *μ*_max_ values for analysts 1-6. Boxes show the interquartile range (IQR), the central line indicates the median, and whiskers represent the 5th–95th percentiles. Outliers outside this range are represented by filled circles. As shown, the spread of calculated mean *μ*_max_ values varied between analysts. **(B)** Spearman correlation heatmap illustrating correlations between the calculated *μ*_max_ values for replicates 1–6 by analysts 1–6. Only replicates 1-6 were used, with the additional replicates present for some strains being excluded. Meaningful correlations |*ρ* | ≥ 0.4 were shown in teal, whilst *ρ* values below the meaningful correlation threshold were shown in different hues of purple.

Broadly speaking, there was a substantial overlap between the values calculated by the scientists. However, the comparative analysis of the *μ*_max_ values calculated by individual researchers also demonstrated considerable differences. The *μ*_max_ values calculated by the analysts referred in this work as #2 and #5 were relatively higher than those calculated by the other four characterised by both higher mean and IQR (Figure 7A). Spearman’s rank correlation coefficients were calculated between replicate runs for each strain across analysts to enable systematic pairwise comparison of *µ*_*max*_ estimate, using only the data from the first six replicate runs for each strain (Figure 7B, Supplementary Table S7). As expected, no meaningful negative correlations were determined (*ρ*_min_ = −0.12), suggesting that valid methodologies were adopted by the researchers and were consistently used across the dataset.

Spearman’s correlation analysis revealed that analysts were not consistently correlated in the *µ*_*max*_ values calculated from replicate runs of the same strains. Analysts #1 and #4 demonstrated greater inter-replicate consistency relative to the remaining analysts. However, this variability may not be attributable solely to analyst-introduced differences; the inherent biological variability between replicate runs likely also influences the decision-making process, with some analysts proving more susceptible to this source of noise than others (Figure 7B, Supplementary Table S7).

A linear mixed-effects modelling approach was used to investigate the relationship between the calculated *μ*_max_ values and the analysts, with *post hoc* pairwise comparisons conducted using estimated marginal means and Tukey-adjusted p-values. A highly significant effect of analyst was observed on calculated *μ*_max_ values (F = 100.6, p = 1.06 × 10^−95^), statistically confirming the results presented above on the substantial analyst-to-analyst variation in the calculation of the maximum specific growth rate. A significant interaction between the strain factor and the analyst (F = 4.42, p < 0.001) indicated that the effect of the human factor on *μ*_max_ calculations varied between strains. Post hoc pairwise comparisons indicated that there was at least one statistically significant difference in *μ*_max_ values (*p* < 0.05) between two analysts for 50% of strains (36/72) investigated in this work.

*H. nigrificans* growth curves led to the highest number of significant differences between the maximum specific growth rate calculations (8 out of 15 pairwise comparisons between analysts). These growth curves were characterised in the AS group, with highly sporadic OD_600_ readings recorded throughout the cultivation (Supplementary Figure S15). However, it would not be entirely true to attribute all analyst-based differences to the nature of the growth curves. *H. gudaonensis*, characterised into the AS group, displayed large variability across replicates, yet this high variability did not yield any significant differences in the determination of the *μ*_max_ values by different analysts (Supplementary Figure S2).

It is widely appreciated that the selection of the data points in the determination of the log-linear range of microbial growth has a large impact on the *μ*_max_ value calculated from the data and subsequently reported in the literature. As an example from the current dataset, the mean *μ*_max_ of *H. hamiltonii* was reported as 0.73 ± 0.15 h^-1^ by Researcher #2 by focussing on the linear range as indicated. Expanding the linear range by including data recorded in an additional 30-min or 60-min timeframe (i.e., including six or 12 additional data points, respectively) ensured that the analysis remained within the linear range, but the resulting mean *μ*_max_ value was calculated as 0.65 ± 0.12 h^-1^ or 0.58 ± 0.09 h^-1^, respectively (Supplementary Figure S16).

**FIGURE 8:**
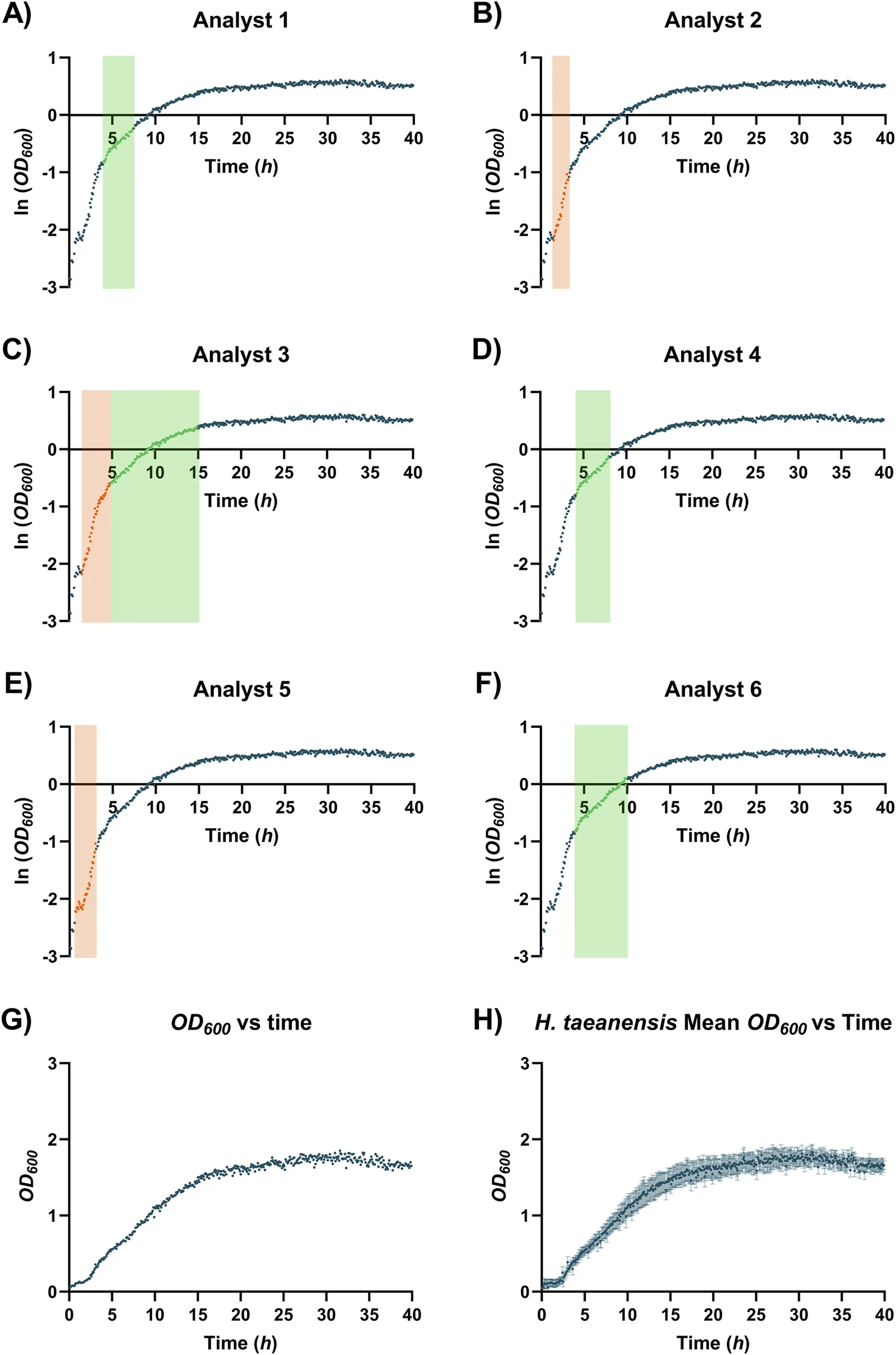
Differences in the selection of the exponential phase of growth between analysts. **(A–F)** Shows the log-transformed growth curve plots for replicate run #4 of *H. taeanensis* cultures. The timeframe selected by each Analysts #1 **(A)**, #2 **(B)**, #3 **(C)**, #4 **(D)**, #5 **(E)**, and #6 **(F)** are shaded in salmon and mint green with the optical density readings in these regions denoted by red and green filled circles, respectively. Orange area with red recordings denotes the initial steep exponential phase and the green shading and data recordings denote the second exponential phase. Panel **(C)** represents the only instance where both phases are used together. Panel **(G)** shows the growth rate of replicate run #4 represented as OD_600_ recordings against time (h). Panel **(H)** shows the mean growth curve of *H. taeanensis* cultures for all replicates (n = 6) represented as mean OD_600_ against time (h). Error bars represent ±1 SD around the mean. *H. taeanensis* was cultured in its recommended growth conditions with *OD*_*600*_ readings recorded every 5 min. For the purpose of this figure, data is only shown up to 40 h.

Likewise, the decisions made by different analysts on the same growth curve directly impacted the calculation of the specific growth rates. To demonstrate this purpose, we focus on the loglinear range selection of the fourth replicate run of *H. taeanensis* by all analysts (Figure 8). The linear range selected for further analysis varies, at times, drastically. This growth curve was particularly interesting as the initial steep linear range was followed by a second linear range indicating the emergence of a second exponential phase of growth before the culture approached stationary phase, which was not classified as DG either. The analysts differed in their reasoning as to how, if at all, these two subsequent exponential phases should be considered in the calculation of the maximum specific growth rate, and how; some considering the initial steep section as the exponential growth phase, whilst others ignoring it in favour of the extended and slower second linear section. Researcher #3, on the other hand, considered both regions separately, reporting a representative mean value at the end. The *μ*_max_ values for this curve were reported as 0.15 h^-1^, 0.68 h^-1^, 0.30 h^-1^, 0.15 h^-1^, 0.51 h^-1^, and 0.14 h^-1^ for Researchers #1 - #6, respectively, exhibiting at times, more than four-fold variability. This exercise offers a clear demonstration of the differences in reported *μ*_max_ values as a result of the subjective interpretations made by researchers in different contexts. Scientist-based variation in *μ*_max_ estimations was not only observed in inter-strain analysis but also, in some cases, inter-replicate analysis of a single strain.

## 4 Discussion

In this study, the growth phenotypes of 82 *Halomonas* strains representing 80 species were systematically characterised under their respective culture collection-recommended conditions, spanning a range of growth media, salt concentrations, and cultivation temperatures. Strains were cultivated in multi-well plates across a minimum of six replicate runs, enabling robust assessment of five qualitative and quantitative growth metrics: growth curve pattern, lag phase duration, maximum specific growth rate, maximum optical density, and time to stationary phase.

The strains displayed considerable diversity in their growth characteristics. Growth profiles were observed to range from well-defined sigmoidal curves to highly erratic profiles. While atypical patterns may reflect intrinsic strain properties, suboptimal cultivation conditions are also known to influence growth phenotypes. This was seen for *H. sediminis* in Figures 3D–F, where changes to the temperature and salt concentration reduced fluctuations and provided more consistent growth patterns. Examples of this have also been seen in other *Halomonas* strains within the literature, with changes in salt concentration and pH affecting the growth phenotype (Kaye and Baross, 2004; Dickinson et al., 2025). In a study by Yu et al., where the effects of salt stress on the physiological metabolomic dynamics of *H. elongata* were investigated, growth patterns were detected when the cells were grown under different salt concentrations (Yu et al., 2022) providing further support to suggest that atypical growth patterns may be affected by suboptimal growth conditions.

Variability in substrate availability may have contributed to the biphasic exponential growth observed in these strains (Figure 8). Stationary phase fluctuations likely reflect stress responses to nutrient depletion, including changes in cell morphology and aggregation (Trunk et al., 2018; Wasim et al., 2009), which could have introduced noise into optical density measurements. At elevated OD values, the linear relationship between optical density and cell density also breaks down (Stevenson et al., 2016; Mira et al., 2022), further complicating stationary phase interpretation.

PHA accumulation during the stationary phase may represent an additional source of optical density fluctuation for *Halomonas* strains. Under conditions of nutrient limitation and elevated cell density, *Halomonas* species are known to accumulate PHA to various degrees as a stress response (Cristea et al., 2018; Garcia-Torreiro et al., 2016). PHA granule accumulation was shown to alter cell morphology and light scattering properties affecting OD_600_ measurements (Martinez and Déziel, 2020).

In their natural environments, bacteria frequently exist within complex and dynamic communities, where conditions and microbial interactions influence growth (Vartoukian et al., 2010; Overmann et al., 2017). Collection recommended conditions are developed to support reliable laboratory cultivation based on physiological characteristics of the strain (Overmann et al., 2017). Here, we opt for these recommended conditions. Whilst assessing growth phenotypes under 27 different cultivation conditions introduces an additional source of complexity in interpretation, the use of recommended conditions can help minimise any unforeseen or unexpected adversities arising from our limited knowledge and understanding of most of these strains. Given the diversity of the 82 strains, in addition to the minimally available literature for many of these, it was not realistic to expect that a single cultivation condition would support all strains equally successfully. Nevertheless, further investigation of these strains under a set of reference cultivation conditions may provide valuable complimentary insights into the growth phenotypes of these strains and further help in identifying strains with potential for future biotechnological applications performing to their optimal capabilities.

The growth curve characterisation can assist understanding the behaviour and cultivation requirements of a strain, and support decision making in the selection of suitable strains for specific applications or in identifying optimum growth conditions. However, it should be noted that the growth curve characterisation is highly subjective. Researchers re-analysing the data at independent times can show differences in classification due to subtleties in the curve patterns impacting the decisions made around classification.

Quantitative growth metrics are needed to understand the *Halomonas* genus comprehensively and successfully identify potential strains for future applications. In this work, this was attempted in the context of a large subset of the genus. Quantitative growth metrics and growth conditions showed little to no meaningful correlations, with the exception of mean *μ*_max_ and mean TtS, which showed a moderately negative correlation, and mean maximum OD_600_ and ${CV}_{{maxOD}_{600}}$ , which showed a strong negative correlation. The former was not unexpected, with faster growing strains reaching stationary phase sooner. However, the latter suggested that strains reaching higher maximum OD_600_ values show less variation, which is an interesting observation that warrants further investigation. The absence of any meaningful correlations between growth metrics and either the recommended temperature or salt concentration suggested that generalisable observations concerning the nutritional, osmolality or cultivation temperature requirements of *Halomonas* cannot be made and is highly strain specific. It should be noted that not observing genome-wide trends does not necessarily exclude the notion that a single strain or a small group of select strains may demonstrate trends in response to varying environmental conditions (Kaye and Baross, 2004; Dickinson et al., 2025).

Historically, fast *μ*_max_ and high cell densities have been attractive phenotypes for bioprocessing. This was particularly prominent in batch or fed-batch fermentation, where rapid growth can help reduce the time of fermentation, allowing for more batches to be run in a given period and higher production rates to be achieved when production was tied to growth (Stanbury et al., 2017). However, for continuous fermentations in next-generation applications, this may not necessarily be the case. Whilst it may still be beneficial to reach the steady state faster, taking slightly longer to reach this point may not be as important in a fermentation that is scheduled to run for weeks or months. Therefore, a relatively slow but robust strain with a consistent performance may be preferable (Olsson et al., 2022). *Halomonas* strains that may show lower *μ*_max_ or TtS but show robust growth and low variability could be ideal candidates for continuous next-generation cultivations.

Based on the quantitative growth metrics, *H. hamiltonii*, *H. zhaodongensis*, *H. heilongjiangensis*, and *H. magadiensis, H. alimentaria*, *H. stevensii*, and *H. venusta* were shortlisted as promising candidates for future biomanufacturing applications. Among those, *H. hamiltonii*, *H. zhaodongensis*, and *H. heilongjiangensis* were identified as the three top-scoring candidates for their quantitative growth metrics. *H. zhaodongensis* and *H. magadiensis* were identified as particularly fast-growing strains, growing at a significantly higher rate than the genus average represented by these 82 strains investigated in this work. The inter-replicate variability of the calculated maximum specific growth rates for *H. venusta* was significantly lower than the genus average, suggesting an exceptionally robust *μ*_max_ for the strain. Consistent and predictable *μ*_max_ allows for easier control of dilution rates in continuous cultivation, enabling a stable cultivation environment close to maximum growth rates at reduced risk of washout.

Except for *H. alimentaria,* all remaining shortlisted strains displayed a mixed qualitative growth characteristic with respect to their growth curve patterns, with different replicate runs displaying S or M shaped growth curve patterns. *H. alimentaria* displayed clear double exponential growth curve characteristics. A possible explanation for this behaviour could be nutrient limitation or the preferential consumption of macronutrient resources, which could manifest as a utilisable characteristic in continuous fermentation.

Of the seven strains shortlisted as promising candidates, most were poorly investigated with respect to their bioprocessing potential. In the literature, only a single study related to bioprocessing applications could be found for each of *H. hamiltonii* and *H. magadiensis*. Suganthi *et al.* investigated the use of *H. hamiltonii* to enhance biodegradation of hydrocarbons in petroleum tank bottom oil sludge (Suganthi et al., 2018), and Cai *et al.* recently investigated the role of appropriately formulated cryoprotection on the performance of *H. magadiensis* for wastewater treatment (Cai et al., 2026). *H. stevensii* was explored for its potential to facilitate CO_2_ fixation either utilising it as a single carbon source or in conjunction with other carbon sources although these studies were confined to specific research groups rather than representing widely explored biotechnological questions (Mishra et al., 2017; Mishra et al., 2018; Khandelwal et al., 2021; Cantera et al., 2022). *H. venusta* was the most studied of the shortlisted strains, with studies reporting a wide range of applications. Examples include PHA production (Stanley et al., 2018), lipopeptide production (Cheffi et al., 2021), and ectoine production (Zhang et al., 2012). No studies related to bioprocessing applications were identified for the remaining three.

Remarkably, *H. zhaodongensis* and *H. hamiltonii* had higher maximum specific growth rates, reached stationary phase faster and achieved higher optical densities than widely studied species of the *Halomonas* genus, such as *H. elongata, H. campaniensis,* and *H. boliviensis*. This further highlights the need for screening studies to help fully characterise a genus and identify the most suitable candidate strains for bioprocessing uses, especially when there is no single type species as a standout candidate for biotechnology applications; incomplete phenotyping of a genus can result in potential candidate strains being missed.

The limited availability of published growth data for non-model and understudied organisms presents a significant challenge for the validation and contextualisation of empirically obtained results. None of the three top-scoring candidate strains identified in this study had previously reported qualitative or quantitative growth characterisation in the literature. For the genus type species, *H. elongata*, however, comparative data are available. Yu et al. reported a *µ*_*max*_ of 0.41 h^-1^ in HE9 synthetic medium containing 6% NaCl (Yu et al., 2025), compared to a mean *µ*_*max*_ of 0.57 ± 0.21 h^-1^ obtained in this study using DSMZ-recommended complex medium (Bacto Marine Broth, 2% NaCl), representing a 39% higher growth rate, though the previously reported *µ*_*max*_ remained within one standard deviation of the replicate mean. This difference is consistent with the well-established tendency of complex media to support higher growth rates relative to defined formulations.

Analysis of inter-analyst variability demonstrated a highly significant effect on *µ*_*max*_ estimations, driven by subjective differences in exponential phase selection. Analysis that prioritised high goodness-of-fit for the linear models tended to produce lower *µ*_*max*_ estimates, likely reflecting more conservative or broader selections that reduce noise but may underestimate the true values. Interestingly, inherent robustness of the growth curves measured by low inter-replicate variability was not a reliable predictor of the magnitude of analyst-induced effects on maximum specific growth rate calculations, suggesting that subtle differences in the growth profile is a prominent factor that drives inter-analyst disagreement. It should be noted that researchers were not given specific instructions as to how to carry out this analysis, so the differences observed are not representative of the human bias for following the same fixed set of instructions, but rather they reflect a much more realistic situation that frequently occurs in research environments, where scientists, with no detailed instructions provided, are just asked to execute an analysis to calculate specific growth rates. We then asked the scientists to provide a ‘short paragraph’ describing their analysis and in return received text with varying levels of depth and detail (see Supplementary Methods). The variability in the outcome coupled with the differences in reporting emphasises the need to provide as much detail as possible regarding the methodology in publications to allow for reproducibility of growth rate calculations by secondary users of data. These results, reinforced by the observations made by Ghenu et al. (2024), highlight the highly subjective nature of growth rate calculations, where both the analytical method employed and researcher-specific interpretation introduce meaningful variability, raising important concerns regarding the reliability and comparability of reported *µ*_*max*_ values across the literature.

While computational methods significantly reduced analysis time, current tools struggled to deal with noisy or atypical growth curves, in some cases misidentifying the exponential phase or yielding disproportionately high *µ*_*max*_ estimates, necessitating additional human oversight. This limitation is particularly relevant for non-model organisms such as *Halomonas*, where atypical growth patterns are common. Developing robust, automated approaches capable of handling such data should therefore be a priority for future work. Standardised computational models would not only accelerate high-throughput analysis but also eliminate researcher-specific bias, providing the consistency and reproducibility that current manual and semi-automated methods cannot reliably deliver.

In conclusion, this study demonstrates the breadth of phenotypic diversity within the *Halomonas* genus and the importance of genus-wide characterisation in identifying high-potential candidate strains that would otherwise be overlooked. The significant inter-analyst variability observed in the calculation of growth parameters, compounded by the limitations of current computational tools in handling atypical growth profiles, highlights the pressing need for standardised, automated analytical frameworks that can deliver the consistency and reproducibility that the field currently lacks. The absence of published growth data for the majority of strains examined further underscores the need for comprehensive, openly accessible phenotypic repositories to support informed strain selection and cross-study comparability. Collectively, these findings advocate for a more systematic and data-driven approach to the characterisation of understudied genera, one that pairs robust cultivation standards with reproducible analytical methods to build the rigorous biological foundation upon which the industrial exploitation of organisms such as *Halomonas* can be reliably advanced.

## Data Availability

The datasets generated and analysed in this study can be found in the UCL Data Repository https://doi.org/10.5522/04/32791977.
