# Supplementary material for: Elucidation of the growth phenotypes of 82 strains from the Halomonas genus
Source: Front Bioeng Biotechnol. 2026 Aug 28;14:1920469. doi: 10.3389/fbioe.2026.1920469 (PMC13562251; doi:10.3389/fbioe.2026.1920469)
Supplement: Supplementary file 1 [file Supplementaryfile1.docx]

Supplementary Material

for

*Elucidation of the growth phenotypes of 82 strains from the Halomonas genus*

Alexander Antoine Maria Baumbach^1^, Waritthorn Thanakarn^1^, André A. B. Coimbra^1^, Iona Wilkinson^1^, Lanqi Zhang^1,2^, Ivano Luigi Colao^1,2^, Duygu Dikicioglu^1*^

^1^Department of Biochemical Engineering, University College London, Gower St, London WC1E 6BT, United Kingdom

^2^Manufacturing Futures Lab, University College London, 7 Sidings Street, London E20 2AE, United Kingdom

# Supplementary Methods: Scientist – specific sections are as described by the analyst with only minimal modifications made to the text to improve clarity

# 1. Growth Rate Calculation Instructions Provided to Scientists

We have collected a large set of growth data for different *Halomonas* strains. There is a separate excel file for each strain. The data shows a Time column in hours (*h*), and subsequent columns showing *OD_600_* values for each replicate. Each strain has at least 6 replicates.

We would like you to use the data provided to calculate the specific growth rates (*µ*) for all strains. A template excel file has been provided where you can enter your calculated *µ* values for the replicates of each strain.

You may plot the growth curves and calculate the *µ* however you like. However, please select the exponential growth periods for *µ* calculations manually. Do not use computational models or line fitting to select the exponential growth phases.

If there are any unclear graphs with difficult to identify exponential growth periods, please add your strategy and reasoning behind how you decided on your exponential growth phase selection in the comment column.

Additionally, please keep your strategy in selecting the exponential growth phases for *µ* calculations consistent for all strains and throughout your data analysis.

Finally, please write a short paragraph outlining how you went about calculating the *µ* in your data analysis.

2. **Method Descriptions Provided by the Scientists**

# 2.1.  Scientist 1

Specific growth rate was determined by first creating additional columns and converting OD_600_ values to ln (OD_600_). The exponential growth phase sections were assessed through visual inspection of both OD_600_ vs Time and ln(OD_600_) vs Time graphs. Using Microsoft Excel, a linear regression line was fitted between 2 chosen time points in the ln(OD_600_) vs Time graphs. The *μ* was then taken as the slope of the curve ln(OD_600_) = m x Time (*h*) + c. R^2^ values for the fitted linear regression was included for each repeat. Ideally, the R^2^ value would be close to 1. In many cases this was not feasible due to the presence of noise in the data.

# 2.2.  Scientist 2

See section 2.3 in main text.

# 2.3.  Scientist 3

The specific growth rate was calculated by identifying the time points of the exponential phase on the growth curve and calculating the gradient between those time points from a natural log plot of the absorbance.

The *OD_600_* vs Time data were uploaded to MATLAB, and the absorbance was plotted against time to obtain the growth curve. The absorbance was also converted to its natural logarithm; these values were then plotted against the corresponding time data in a separate plot. A minor grid was added to the plot for better numerical observation.

The exponential phase was identified as the section of the growth curve where an exponential increase in absorbance occurred. Slow increases at the beginning or end of the exponential portion were determined as part of the lag and death phases and were not considered. The 2 points at the beginning and end of the exponential phase were manually chosen, and their x-values were noted. On the logarithmic plot, points with the same x values were chosen, and these points were used to calculate the gradient between those points. The identified gradient was taken as the specific growth rate.

If two possible exponential phases were present, the initial phase was selected. However, if this phase was less than ¼ of the overall growth phase, the growth rate was calculated for both selections and the mean of both phases was taken.

# 2.4.  Scientist 4

First, each OD_600_ data was calculated to ln(OD_600_) for each time point. Then, the graph was plotted by ln(OD_600_) and time for y and x axis, respectively from time 0 hour until the end of data set. After that, the graph was shortened only for linear section (data was plotted approximately more than 2 hours). R^2^ value was calculated for the specific selection. Growth rates were taken from the slope of linear equation or in the following equation, y=ax+b. where R^2^ was more than 0.9. The data set from each replicate might not be the same time series if R^2^ value was less than 0.9. Then, that replicate data was adjusted individually until R^2^ was more than 0.8-0.9.

# 2.5.  Scientist 5

Analysis was conducted using Python version 3.13.5, with the Pandas version 2.2.3 (The pandas development team, 2024, McKinney, 2010), NumPy version 2.1.3 (Harris et al., 2020), and SciPy version 1.15.3 (Virtanen et al., 2020) packages.

First, data files were imported into Python, and OD_600_ values less than or equal to 0 were replaced with 1 x 10^-6^ to allow for ln (OD_600_) transformations. To reduce the noise of the data, 12-point rolling mean was then calculated to smooth the curves. Using the formula *μ* = (ln(Xt) − ln(X0)) / (Tt − T0), *μ* was calculated for every possible time window and starting from every possible starting time using the smoothed curve, i.e., calculating every possible *μ* value across the smoothed growth curve. Additionally, an R^2^ value was calculated for each time window.

To filter the data, only the window with R^2^ > 0.95 were kept confirming linear exponential growth. Next, for each starting time point, only the window with the maximum *μ* value was kept. Δt = End Hour – Start Hour and Δt x *μ* values were created for these time windows and calculated *μ_max_* Values. Only windows with Δt ≥ 1 hour were kept to further filter out any non-meaningful time windows. Finally, the time window with the largest Δt x *μ* was selected for the exponential growth phase, and the corresponding *μ* was taken as the *μ_max_.*

# 2.6.  Scientist 6

For all datasets of online optical density (OD_600_) provided the following method and assumptions were used to determine the value of the maximum cell growth constant (*µ_max_*) by Investigator X.

For each raw dataset provided, Microsoft Excel was used for all graphing, computation and data handling/manipulation. Initially, a full-process growth curve of OD_600_ versus process time was plotted to determine the overall growth mechanics of the cells (a new graph being generated for each individual replicate), identify any non-standard growth parameters/assay-derived noise/fluctuations and to determine an expected linear region (n.b. this linear region as determined by OD_600_ vs Time was only used qualitatively to set expectation of data ranges, and not as the final method to derive µ_max_ itself.)

Where the online data showed double growth curves, an assumption was made that only the first of these curves would be analysed* as this is a) the most relevant curve for the process type in question, and b) that the second curve was more likely to be generated by metabolic shift (e.g. change of C-source being utilised by the cells), or some other influencing phenomenon which would be eradicated by process optimisation and the ability to control feeds which were not possible within a microwell plate during an initial screening study.

Upon visualisation of the entire curve of OD_600_ vs time, the investigator would then take the natural logarithm of the OD_600_ values and plot them in the established semi-log plot method against time for the entire process. Iteratively, time points would be removed to remove the stationary and death phases, and (if applicable) the initial lag phase of each culture to yield a straight-line graph denoting the [first*, if applicable] exponential phase of culture. As an added check, the investigator compared the predicted linear region with the original OD_600_ vs Time graph to ensure the regions were appropriately matched between observation and expectation. A similar check between datasets was performed with all OD_600_ vs Time graphs being compared visually (i.e. OD_600_ vs Time for all individual replicates within a single strain’s raw data file), to ensure that the data being processed was not a copy of a previous replicate/strain.

In addition, where the initial values of OD_600_ (and thus ln (OD_600_) were highly variable due to initial instability of measurements derived from the assay itself, the plot of ln (OD_600_) would also be redacted (reasonably) from the leading end to minimise this initial fluctuation. A similar task was performed for fluctuations which occurred at the end of the linear region (i.e. in the late-exponential leading to stationary phase of culture.) If within the full duration of the exponential phase a continuous gradient shift was observed mid-way and which skewed one half of the phase from the other, the greatest gradient was selected to determine *μ_max_*.

N.B. In compliance with the first author’s instructions, no manipulation/redaction of data was performed within the linear range even if noise/fluctuations were observed although it was noted by the investigator that improvements to the method could have been made by use of noise reduction methods such as moving averages or reduction of plotted data points to account for larger gaps of time (i.e. evenly spread data skipping, e.g. picking only points every 5 minutes vs 30 seconds) where the change in O1D_600_ would potentially outweigh the individual point-to-point fluctuations given by the measurement method.

Once a final linear region had been identified across each replicate (typically noted as beginning around 2-4 hours and ending between 8-16 hours) the investigator would use curve fitting to produce a linear line of best fit (LOBF). Once completed, the LOBF’s linear equation was generated by excel in the form of y = mx + c, from which the value of µmax was determined as equal to the value of the gradient, m, and reported in a summary table, alongside visual observations and comments of the overall growth curve, and the stability/variability of the trends therein.

# Supplementary Figures

**Supplementary Figure S1.** Growth curves of *H. johnsoniae* replicates*. H. johnsoniae* was grown in its recommended growth conditions with optical density at 600nm (OD_600_) recorded every 5 minutes. Panels A-F represent the six replicate runs.

**Supplementary Figure S2.** Growth curves of *H. gudaonensis* replicates*. H. gudaonensis* was grown in its recommended growth conditions with optical density at 600nm (OD_600_) recorded every 5 minutes. Panels A-F represent the six replicate runs.

**Supplementary Figure S3.** Growth curves of *H. songnenensis* replicates*. H. songnenensis* was cultured under its recommended growth conditions with optical density at 600nm (OD_600_) recorded every 5 minutes. Panels A-F represent the six replicate runs.

**Supplementary Figure S4.** Growth curves of *H. sediminis* replicates cultured at 10% salt concentration and 37°C*. H. sediminis* was cultured at 37°C in its recommended growth medium with a final salt concentration of 10% (w/v). Optical density at 600nm (OD_600_) was recorded every 5 minutes. Panels A-F represent the six replicate runs.

**Supplementary Figure S5.** Growth curves of *H. sediminis* replicates cultured at *10*% salt concentration and 28°C*. H. sediminis* was cultured at 28°C in its recommended growth medium with a final salt concentration of 10% (w/v). Optical density at 600nm (OD_600_) was recorded every 5 minutes. Panels A-F represent the six replicate runs.

**Supplementary Figure S6.** Growth curves of *H. sediminis* replicates cultured at 2% salt concentration and 28°C*. H. sediminis* was cultured at 28°C in its recommended growth media with a final salt concentration of 2% (w/v). Optical density at 600nm (OD_600_) was recorded every 5 minutes. Panels A-F represent the six replicate runs.

**Supplementary Figure S7.** Growth curves of *H. hamiltonii* replicates*. H. hamiltonii* was cultured under its recommended growth conditions with optical density at 600nm (OD_600_) recorded every 5 minutes. Panels A-F represent the six replicate runs.

**Supplementary Figure S8.** Growth curves of *H. zhaodongensis* replicates*. H. zhaodongensis* was cultured under its recommended growth conditions with optical density at 600nm (OD_600_) recorded every 5 minutes. Panels A-F represent the six replicate runs.

**Supplementary Figure S9.** Growth curves of *H. heilongjiangensis* replicates*. H. heilongjiangensis* was cultured under its recommended growth conditions with optical density at 600nm (OD_600_) recorded every 5 minutes. Panels A-F represent the six replicate runs.

**Supplementary Figure S10.** Growth curves of *H. magadiensis* replicates*. H. magadiensis* was cultured under its recommended growth conditions with optical density at 600nm (OD_600_) recorded every 5 minutes. Panels A-F represent the six replicate runs.

**Supplementary Figure S11.** Growth curves of *H. alimentaria* replicates*. H. alimentaria* was cultured under its recommended growth conditions with optical density at 600nm (OD_600_) recorded every 5 minutes. Panels A-F represent the six replicate runs.

**Supplementary Figure S12.** Growth curves of *H. stevensii* replicates*. H. stevensii* was cultured under its recommended growth conditions with optical density at 600nm (OD_600_) recorded every 5 minutes. Panels A-F represent the six replicate runs.

**Supplementary Figure S13.** Growth curves of *H. venusta* replicates*. H. venusta* was cultured under its recommended growth conditions with optical density at 600nm (OD_600_) recorded every 5 minutes. Panels A-F represent the six replicate runs.

**Supplementary Figure S14.** Growth curves of *H. subglaciescola* replicates showing the exponential growth phase selections by Analyst 5 for each repeat. *H. subglaciescola* was cultured under its recommended growth conditions with optical density at 600nm (*OD_600_*) recorded every 10 minutes to allow for extended growth. Panels A-F represent the six replicate runs. The exponential growth phase selection is shown by the orange dots and highlighted orange rectangle.

**Supplementary Figure S15.** Growth curves of replicates 1-6 of *H. nigrificans. H. nigrificans* was cultured under its recommended growth conditions with optical density at 600nm (*OD_600_*) recorded every 5 minutes. Panels A-F represent the six replicate runs. Repeats 7-24 not included in this Figure.

**Supplementary Figure S16.** Figure showing the log transformed growth curves of the six replicate runs of *H. hamiltonii* for the initial 15 h of the cultures*.* The exponential growth phase selection is shown by the orange dots and highlighted salmon rectangle. The inclusion of another 30 or 60 minutes are marked by the mint and lilac shaded regions. *H. hamiltonii* was cultured under its recommended growth conditions with OD_600_ readings taken every 5 minutes. For the purposes of this Figure, data only up to 40 hours is shown.

# References

HARRIS, C. R., MILLMAN, K. J., VAN DER WALT, S. J., GOMMERS, R., VIRTANEN, P., COURNAPEAU, D., WIESER, E., TAYLOR, J., BERG, S., SMITH, N. J., KERN, R., PICUS, M., HOYER, S., VAN KERKWIJK, M. H., BRETT, M., HALDANE, A., DEL RÍO, J. F., WIEBE, M., PETERSON, P., GÉRARD-MARCHANT, P., SHEPPARD, K., REDDY, T., WECKESSER, W., ABBASI, H., GOHLKE, C. & OLIPHANT, T. E. 2020. Array programming with NumPy. *Nature,* 585**,** 357-362.

MCKINNEY, W. 2010. Data Structures for Statistical Computing in Python. *SciPy 2010*.

THE PANDAS DEVELOPMENT TEAM 2024. pandas-dev/pandas: Pandas. v2.2.3 ed.: Zenodo.

VIRTANEN, P., GOMMERS, R., OLIPHANT, T. E., HABERLAND, M., REDDY, T., COURNAPEAU, D., BUROVSKI, E., PETERSON, P., WECKESSER, W., BRIGHT, J., VAN DER WALT, S. J., BRETT, M., WILSON, J., MILLMAN, K. J., MAYOROV, N., NELSON, A. R. J., JONES, E., KERN, R., LARSON, E., CAREY, C. J., POLAT, İ., FENG, Y., MOORE, E. W., VANDERPLAS, J., LAXALDE, D., PERKTOLD, J., CIMRMAN, R., HENRIKSEN, I., QUINTERO, E. A., HARRIS, C. R., ARCHIBALD, A. M., RIBEIRO, A. H., PEDREGOSA, F., VAN MULBREGT, P., VIJAYKUMAR, A., BARDELLI, A. P., ROTHBERG, A., HILBOLL, A., KLOECKNER, A., SCOPATZ, A., LEE, A., ROKEM, A., WOODS, C. N., FULTON, C., MASSON, C., HÄGGSTRÖM, C., FITZGERALD, C., NICHOLSON, D. A., HAGEN, D. R., PASECHNIK, D. V., OLIVETTI, E., MARTIN, E., WIESER, E., SILVA, F., LENDERS, F., WILHELM, F., YOUNG, G., PRICE, G. A., INGOLD, G.-L., ALLEN, G. E., LEE, G. R., AUDREN, H., PROBST, I., DIETRICH, J. P., SILTERRA, J., WEBBER, J. T., SLAVIČ, J., NOTHMAN, J., BUCHNER, J., KULICK, J., SCHÖNBERGER, J. L., DE MIRANDA CARDOSO, J. V., REIMER, J., HARRINGTON, J., RODRÍGUEZ, J. L. C., NUNEZ-IGLESIAS, J., KUCZYNSKI, J., TRITZ, K., THOMA, M., NEWVILLE, M., KÜMMERER, M., BOLINGBROKE, M., TARTRE, M., PAK, M., SMITH, N. J., NOWACZYK, N., SHEBANOV, N., PAVLYK, O., BRODTKORB, P. A., LEE, P., MCGIBBON, R. T., FELDBAUER, R., LEWIS, S., TYGIER, S., SIEVERT, S., VIGNA, S., PETERSON, S., MORE, S., PUDLIK, T., OSHIMA, T., et al. 2020. SciPy 1.0: fundamental algorithms for scientific computing in Python. *Nature Methods,* 17**,** 261-272.
